# Regulation of biofilm formation in *Klebsiella pneumoniae*

**DOI:** 10.3389/fmicb.2023.1238482

**Published:** 2023-09-07

**Authors:** Yin Li, Ming Ni

**Affiliations:** Department and Institute of Infectious Disease, Tongji Hospital, Tongji Medical College and State Key Laboratory for Diagnosis and Treatment of Severe Zoonotic Infectious Disease, Huazhong University of Science and Technology, Wuhan, China

**Keywords:** *Klebsiella pneumoniae*, biofilm, capsule, fimbriae, factors

## Abstract

*Klebsiella pneumoniae* is an important Gram-negative opportunistic pathogen that is responsible for a variety of nosocomial and community-acquired infections. *Klebsiella pneumoniae* has become a major public health issue owing to the rapid global spread of extensively-drug resistant (XDR) and hypervirulent strains. Biofilm formation is an important virulence trait of *K. pneumoniae*. A biofilm is an aggregate of microorganisms attached to an inert or living surface by a self-produced exo-polymeric matrix that includes proteins, polysaccharides and extracellular DNA. Bacteria within the biofilm are shielded from antibiotics treatments and host immune responses, making it more difficult to eradicate *K. pneumoniae*-induced infection. However, the detailed mechanisms of biofilm formation in *K. pneumoniae* are still not clear. Here, we review the factors involved in the biofilm formation of *K. pneumoniae*, which might provide new clues to address this clinical challenge.

## 1. Introduction

*Klebsiella pneumoniae* is an important pathogenic Gram-negative, nonmotile bacterium that is responsible for a variety of common infections including urinary tract infections (UTIs), pneumonia, bacteremia, purulent liver abscesses, and wound infection (Karyoute, 1989; Bouza et al., 2001; Luo et al., 2014; Mohamudha et al., 2016; Bart et al., 2021; D’Abbondanza and Shahrokhi, 2021; GBD 2019 Antimicrobial Resistance Collaborators, 2022). Given the rapid spread of extensively-drug resistant (XDR) (mainly carbapenem resistant *K. pneumoniae* [CRKP]) and hypervirulent strains worldwide, *K. pneumoniae* has become a major problem for public health. In 2020, the Antimicrobial Testing Leadership and Surveillance (ATLAS) program collected a total of 6,753 *K. pneumoniae* isolates from 57 countries across six regions worldwide. Of these isolates, 1,118 (16.6%) were CRKP strains (Lee et al., 2022). Because of the limitation of treatment options, CRKP is considered an urgent clinical threat. It was estimated that antibiotic resistant *K. pneumoniae* was responsible for more than 600,000 deaths globally in 2019 (Antimicrobial Resistance Collaborators, 2022). Hypervirulent *K. pneumoniae* (hvKP), a more virulent evolving variant of *K. pneumoniae*, is known to cause community-acquired, metastatic, and life-threatening infections such as pyogenic liver abscesses (PLA), central nervous system infection and endophthalmitis, which require rapid recognition and site-specific treatment (Russo and Marr, 2019). Moreover, via acquisition of carbapenem-resistant plasmids or hvKP-specific virulence determinants, XDR-hvKP strains are emerging (Han et al., 2022; Tian et al., 2022). The prevalence of the XDR-hvKP and its potential threat to human health are of concern.

Biofilm formation is an important virulence trait for *K. pneumoniae* (Khodadadian et al., 2018; Tang et al., 2020; Shadkam et al., 2021). A biofilm is a type of polymerization wherein microorganisms attach to inert or active surfaces through the extracellular polymeric substances (EPS) that they produce (Ashwath et al., 2022). The EPS is largely composed of polysaccharides, proteins, nucleic acids, lipids, and extracellular DNA (eDNA) (Bertoglio et al., 2018). The polysaccharides of *K. pneumoniae* biofilms are composed of mannose, glucose, their amines, and acetylated counterparts. The expression of the proteins in biofilms are different owing to the heterogeneity of the environment (Singh et al., 2019). Although the kinetics of biofilm formation vary among different strains, the process of biofilm formation is similar, including initial attachment, micro-colony formation, maturation, and dispersion (Bandeira et al., 2014; Koo et al., 2017; Al-Bayati and Samarasinghe, 2022). Each step requires many bacterial functions, such as exercise, adhesion, transportation, stress response, metabolic pathway activation, and extracellular matrix synthesis (Beloin et al., 2004; Domka et al., 2007).

The overall properties of biofilms are endowed by the biofilm matrix and can protect the resident cells against desiccation, chemical interference and other bacterial invasion. Moreover, biofilms help bacterial cells resist killing by the human phagocytic system, and they ensure that the biofilm community continues to adhere to the media surface (Yan and Bassler, 2019). It was estimated that the resistance of bacterial biofilm to antimicrobial agents might be 10–1,000-times higher than planktonic bacteria (de la Fuente-Núñez et al., 2016). Additionally, the bacteria in these fixed communities are often in close proximity to one another, which also increases the probability of chemical signal transduction and gene transfer between bacterial cells of the same or different species (Cook and Dunny, 2014; Koraimann and Wagner, 2014). This provides more convenient conditions for the spread of drug resistance genes. A previous report suggested that 65–80% of bacterial infections had some connection with biofilm formation (Al-Bayati and Samarasinghe, 2022). In patients with recurrent infections, it was observed that most serial isolates recovered from these patients were strong biofilm producers *in vitro* (Sanchez et al., 2013).

Nosocomial infections caused by *K. pneumoniae* are facilitated by the ability of the organism to form biofilms (Bandeira et al., 2014). It was reported that the majority of clinically isolated *K. pneumoniae* formed biofilms. Among the isolated *K. pneumoniae* strains, 54%, 29%, and 14% were strong, moderate and weak biofilm producers, respectively. The major sources of strong biofilm producers were isolated from urine, pus, and blood, whereas moderate biofilm producers were isolated from blood (Ashwath et al., 2022).

This review focuses on *K. pneumoniae* biofilms and summarizes various factors and genes affecting biofilm formation in *K. pneumoniae*.

## 2. Major factors affecting biofilm formation of *Klebsiella pneumoniae*

### 2.1. Capsule

The capsule affects different stages of biofilm formation of *K. pneumoniae*. It controls the initial adhesion through a series of behaviors, such as improving the regular initial spatial distribution and preventing bacterial interactions as much as possible (Balestrino et al., 2008). Davis et al. pointed out that the capsule was necessary for constructing an appropriate initial covering of mature biofilm structure (Davis and Brown, 2020). The expression of capsular polysaccharide also ensures that *K. pneumoniae* forms a typical three-dimensional mature biofilm structure (Balestrino et al., 2008).

The capsule is a toxic bacterial component. So far, researchers have identified 134 capsule synthesis loci (K loci) (Wyres et al., 2016). In a study using signature-tagged mutagenesis (STM) to screen a *K. pneumoniae* mutant library with unique characteristic markers to identify genes related to biofilm formation, the authors found that mutations in capsule gene cluster sites could lead to defects in biofilm formation (Boddicker et al., 2006). Mutants that insert transposons in the capsular *wza* and *wzc* loci show defective biofilm formation (Wu et al., 2011). Balestrino et al. (2008) discovered that the biofilm forming ability of *K. pneumoniae* ORF4 (*wza* homologous, transport of capsular polysaccharides) and ORF14 (glycosyl transferase, capsule biosynthesis) mutants on polyvinyl-chloride (PVC) was significantly reduced.

Further, *treC* and *sugE* have been shown to affect biofilm formation of *K. pneumoniae* by regulating the production of capsular polysaccharide (CPS). *TreC* encodes trehalose-6-phosphate hydrolase, and its deletion affects bacterial utilization of trehalose. *TreC* mutants show reduced mucus viscosity and produce less CPS, thereby reducing biofilm formation and preventing formation of advanced biofilm structures in *K. pneumoniae*. Adding glucose to the culture medium of *K. pneumoniae treC* mutant strains can restore CPS production and biofilm formation. However, a *sugE* (encoding an intima protein) mutant that increases biofilm formation in *K. pneumoniae* shows higher mucus viscosity and produces more CPS. It was suggested that the absence of *sugE* in *K. pneumoniae* would lead to changes in bacterial membrane structure and activate the downstream cascade, thus increasing CPS production during biofilm formation (Wu et al., 2011).

Biofilm formation in isolates containing *magA* (K1), *rmpA*+, *rmpA2*+, the virulence factors related to capsule production, is more obvious than in isolates with negative virulence factors. However, multivariate regression analysis showed that *wcaG* was the only independent risk factor for biofilm (Zheng et al., 2018). The *wcaG* positive genotype was involved in K1 and K54 capsular types, and was less associated with K16 and K58 capsular types (Turton et al., 2010). In addition, *wcaG* encodes the protein participating in the biosynthesis of fucose, and the deletion mutation of *wcaG* affects most capsule polysaccharide genes (Ho et al., 2011), thereby suggesting that *wcaG* may affect biofilm formation by changing the composition of capsule polysaccharides (Zheng et al., 2018).

One study also showed that the capsule could inhibit biofilm formation in *K. pneumoniae*. A recent study showed that strains lacking the *wbaP* gene, which was related to capsule production, formed stronger biofilms. While the strains with the super capsule containing *wzc* mutation could not form biofilms (Ernst et al., 2020). When carbohydrates were added to the medium, the biosynthesis of CPS increased, but the biofilm formation in *K. pneumoniae* decreased (Chen et al., 2020). A previous study showed that the expression of CPS in *K. pneumoniae* physically interrupted the function of type 1 fimbriae, hindered the biofilm formation mediated by fimbriae, and reduced the adhesion of bacteria to the surface (Schembri et al., 2005). In addition, CPS could inhibit bacterial surface interactions on non-biological substrates (dos Santos Goncalves et al., 2014). It was found that the capsule was costly in nutrient rich media, but it provided obvious adaptive advantages under conditions of malnutrition. Further, among strains forming more biofilms, the capsule often played a positive role in biofilm formation. The authors suggested that this was not because of the presence or absence of capsules, but was instead caused by the amount of capsule expressed by a given strain, which then affected biofilm formation. Moreover, the function of the capsule was not conservative in different isolates, but relied on other elements of the genome or serotype (Buffet et al., 1946).

The mechanism by which the capsule influences biofilm formation and the conditions under which it has positive or negative regulation on biofilm formation are still unclear. However, there seems to be a relationship with the O antigen, because the polysaccharide capsule is retained on the outer surface of the bacteria by interacting with the repeat sugar molecule of the lipopolysaccharide (LPS) molecule, namely “O antigen.” The *waaL* gene encodes a ligase involved in the connection of the LPS repeat O antigen to the LPS core, and inactivation of this gene is understood to lead to a significant reduction in capsule retention and an increase in biofilm formation (Singh et al., 2022).

### 2.2. Fimbriae

The *K. pneumoniae* genome encodes for several types of fimbriae. Fimbriae are hair-like protein appendages extending from the cell surface (Wilksch et al., 2011). Fimbriae promote *K. pneumoniae* adhesion to non-biological surfaces, resulting in catheter related infections (Schroll et al., 2010). Type 1 and type 3 fimbriae, the most studied fimbriae, are encoded by *fim* and *mrk* gene clusters, respectively. In addition, *ecp* and *kpa* to *kpg* gene clusters found in recent years also encode fimbriae (Wu et al., 2010; Alcántar-Curiel et al., 2013).

In *K. pneumoniae*, biofilm formation is mainly mediated by type 3 fimbriae, and the Mrk protein is encoded by the operon containing the *mrkABCDF* gene (Allen et al., 1991). Type 3 fimbriae are mainly made up of the main fimbriae subunit, MrkA, which polymerizes to form a spiral fimbriae axis (Murphy and Clegg, 2012). In addition, Δ*mrkA* mutants are unable to attach to the abiotic surface to form biofilms (Di Martino et al., 2003; Jagnow and Clegg, 2003). Further, MrkA protein expression is significantly upregulated during biofilm thickening (Vuotto et al., 2017). MrkB and MrkC have sequence characteristics representing the periplasmic chaperone and usher translocatase, respectively. MrkD, present on the top of the fimbriae surface, also has adhesion characteristics of appendages, and determines specificity of fimbriae binding (Murphy and Clegg, 2012). The *mrkA* and *mrkD* genes play a key role in the biofilm formation of *K. pneumoniae* (Fang et al., 2021). The *mrkA* gene contributes to rapid biofilm formation while *mrkD* contributes to form dense *K. pneumoniae* biofilms (Ashwath et al., 2022). A gene cluster, *mrkHIJ*, adjacent to the type 3 fimbriae operon is related to the regulation of type 3 fimbriae expression. *MrkH* is a new transcription activator of the *mrk* gene cluster, which regulate *mrkHI* expression and contains the PilZ domain. *MrkH* binds to the region upstream of the *mrkA* promoter and activates the expression of the *mrkABCDF* operon. Therefore, *mrkH* is often referred to as a “biofilm switch” as it can initiate expression of genes involved in producing type 3 fimbriae (Wilksch et al., 2011; Tan et al., 2015). The biofilm formation capacity of *K. pneumoniae* carrying the *mrkH* box was clearly higher than strains without it (Fang et al., 2021). Wu et al. (2012) found that *mrkHI* transcription could be activated by *MrkI*. *MrkI* is a LuxR-like regulatory factor. The *mrkI* mutant reduces the mannose-resistant *Klebsiella*-like (MR/K) hemagglutinins (HA) activity and the number of type 3 fimbriae on the cell surface, leading to a significant reduction in biofilm formation, which can be rescued when providing wild type *mrkI* copies (Johnson et al., 2011; Wilksch et al., 2011). The expression of *mrkHI* is also actively regulated by Fur, which usually acts as a transcriptional activator to directly activate the transcription of *mrkHI*. The deletion of Fur reduces *mrkH*, *mrkI,* and *mrkA* transcription, thereby reducing type 3 fimbriae expression and biofilm formation (Wu et al., 2012). In addition, at least two components of pulmonary surfactant, phosphatidylcholine and cholesterol, promote the transcription of type 3 fimbriae genes and biofilm formation of *K. pneumoniae* (Willsey et al., 2018).

The type 3 fimbriae-dependent adhesion is probably the initial stage of *K. pneumoniae* colonization and biofilm formation on non-biological surfaces (Duguid, 1959). Type 3 fimbriae mediate the binding to the surface of damaged epithelium as they can bind to the extracellular matrix of urinary and respiratory tissues (Tamayo et al., 2007).

Type 3 fimbriae not only participate during the initial stages of *K. pneumoniae* biofilm formation, but also mediate the c-di-GMP dependent bacterial growth mode transformation from planktonic to biofilm. c-di-GMP is an important second messenger in bacteria (Tamayo et al., 2007). The activity of diguanylate cyclase (DGC) and phosphodiesterase (PDE) can regulate the intracellular concentration of c-di-GMP in bacteria (Simm et al., 2004; Hengge, 2009). The *mrkHIJ* gene cluster is associated with the regulation and sensing of c-di-GMP (Lin et al., 2016). When activated by c-di-GMP, MrkH recruits RNA polymerase to the *mrkHI* promoter to auto-activate *mrkH* expression. Increased MrkH production subsequently drives the expression of *mrkABCDF*, leading to type 3 fimbriae biosynthesis and biofilm formation (Tan et al., 2015). *MrkJ* encodes a hypothetical phosphodiesterase (PDE) which contains an EAL domain (the sequences encoding diguanylate cyclase and phosphodiesterase A share a lengthy consensus motif, comprising two adjacent domains termed GGDEF and EAL) mediating the hydrolysis of c-di-GMP (Tal et al., 1998; Johnson and Clegg, 2010). Because of intracellular accumulation of c-di-GMP, the absence of *mrkJ* leads to an increase in the production of type 3 fimbriae and biofilm formation (Johnson and Clegg, 2010; Wilksch et al., 2011). *YjcC* possesses PDE activity in the recombinant protein of its EAL domain. After receiving oxidative stress signal input, YjcC actively regulates oxidative stress responses by changing the level of c-di-GMP and has a negative impact on type 3 fimbriae expression and biofilm formation (Huang et al., 2013). YfiN harbors DGC domain plays a positive role in the expression of type 3 fimbriae (Wilksch et al., 2011). OmpR/EnvZ is a two-component system that senses osmotic signals and controls downstream gene expression in many species of *Enterobacteriaceae*. In response to osmotic stresses, the phosphorylated form of OmpR of *K. pneumoniae* regulates the expression of type 3 fimbriae to influence biofilm formation via modulating the level of intracellular c-di-GMP and MrkHIJ (Lin et al., 2018).

**Figure 1:**
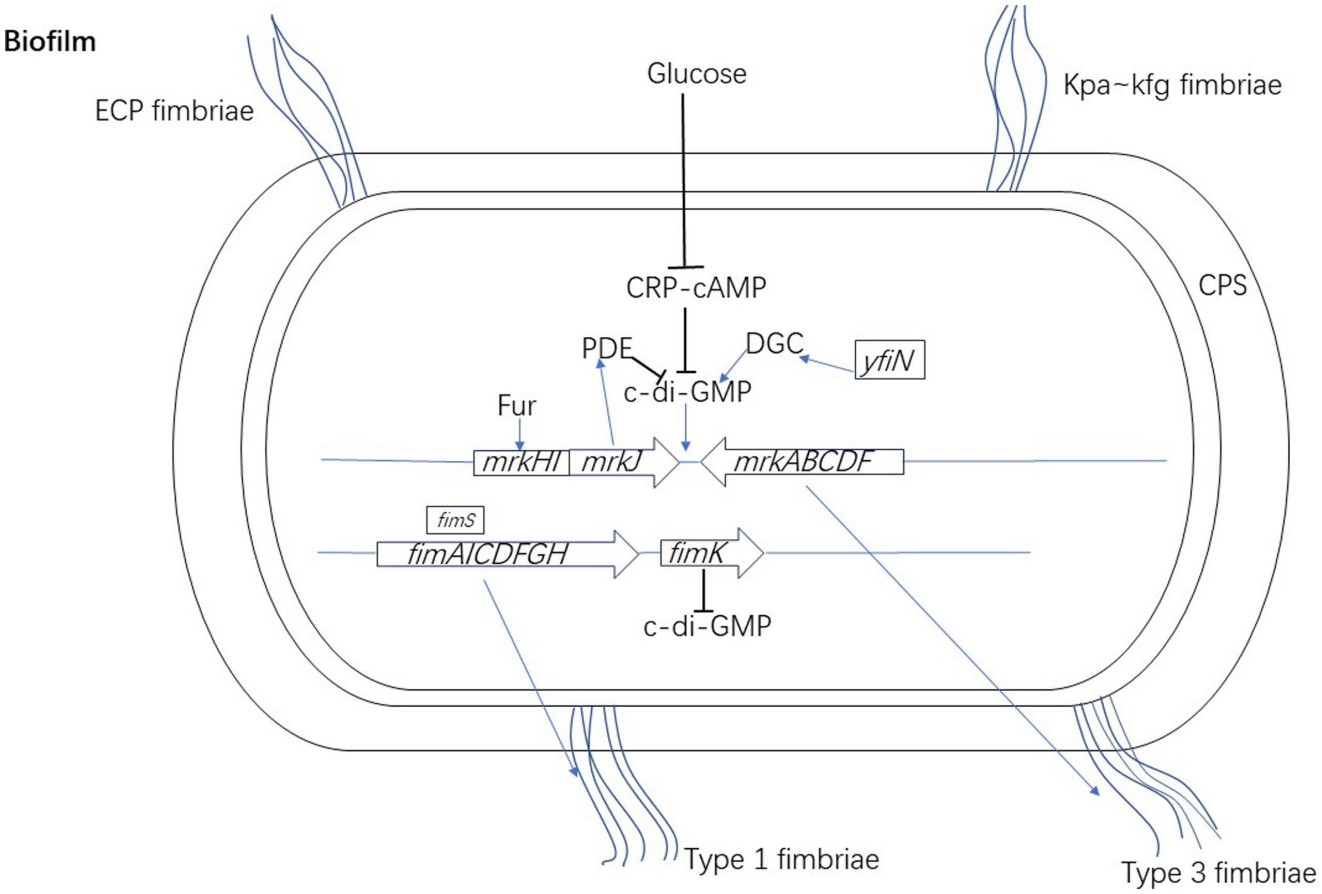
The regulation mechanisms of the biosynthesis of type 3 and type 1 fimbriae of *K. pneumoniae.* When activated by c-di-GMP, MrkH recruits RNA polymerase to the *mrkHI* promoter to auto-activate *mrkH* expression. Increased MrkH production subsequently drives the expression of *mrkABCDF*, leading to type 3 fimbriae biosynthesis and biofilm formation. The expression of *mrkHI* is also actively regulated by Fur, which usually acts as a transcriptional activator to directly activate the transcription of *mrkHI*. *MrkJ* encodes PDE which contains an EAL domain mediating the hydrolysis of c-di-GMP. YfiN harbors DGC domain plays a positive role in the expression of type 3 fimbriae by changing the level of c-di-GMP. CRP-cAMP indirectly regulates the expression of type 3 fimbriae through inhibiting the c-di-GMP signal pathway. Type 1 fimbriae are encoded by the *fim* gene cluster. The regulation of *fim* gene expression is controlled by *fimS*. The regulatory gene *fimK* constitutes the *fim* operon, and FimK can regulate intracellular levels of c-di-GMP.

Type 1 fimbriae, also known as mannose sensitive fimbriae, can bind soluble mannose as a competitive inhibitor, as the name suggests. Type 1 fimbriae are encoded by the *fim* gene cluster, which is composed of eight genes (*fimAICDFGHK*) (Gomes et al., 2020). Regulation of *fim* gene expression is controlled by reversible DNA elements (*fimS*). Type 1 fimbriae are composed of a major fimbriae subunit, FimA, and a minor apical adhesion protein, FimH (Alcántar-Curiel et al., 2013). FimH is a component that promotes adhesion to the host surface and contains mannose (Sahly et al., 2008). The increased expression of the *fimH* gene plays an important role in the binding of bacteria to surfaces, leading to strong biofilm formation (Ashwath et al., 2022). The regulatory gene *fimK* constitutes the *fim* operon, and FimK has an EAL domain with PDE characteristics, which can regulate intracellular levels of c-di-GMP (Johnson and Clegg, 2010). Mutant strains that cannot produce FimK have a higher fimbriate shape than wild type *K. pneumoniae* and can be planted in the urinary tract of mice (Johnson and Clegg, 2010). FimK can reduce type 1 fimbriae, and inhibit biofilm formation and intracellular bacterial communities (Rosen et al., 2008). Type 1 fimbriae are key causes of UTIs (Struve et al., 2008) and they have high affinity of mannose residues on bladder cell surfaces (Rozen and Skaletsky, 2000), which can promote the adhesion and invasion of epithelial bladder cells, thus forming biofilm-like intracellular bacterial communities (Rosen et al., 2008). The regulation mechanisms of the biosynthesis of type 3 and type 1 fimbriae are shown in Figure 1.

Type 1 and type 3 fimbriae can contribute to biofilm formation and compensate for each other (Stahlhut et al., 2012; Murphy et al., 2013; Ashwath et al., 2022). One study using the catheter bladder model, found that type 1 and type 3 fimbriae enhance biofilm formation on catheters (Stahlhut et al., 2012). Moreover, it was reported that gene clusters of type 3 and type 1 fimbriae have a cross regulatory effect, and the up-regulation of type 1 fimbriae can make up for the loss of type 3 fimbriae expression (Schroll et al., 2010). Type 3 fimbriae may have a more significant effect on biofilm formation than type 1 fimbriae (Fang et al., 2021). Bacterial strains that cannot produce type 1 fimbriae are as proficient as bacterial strains that can produce such fimbriae in biofilm formation (Clegg and Murphy, 2016).

Alcantar-Curiel et al. found that except for type 3 and type 1 fimbriae, the *Escherichia coli* common pilus (ECP) fimbriae gene cluster in the *K. pneumoniae* genome has an operon that is homologous to the *E. coli* ECP fimbriae. The ECP fimbriae gene cluster contains the *ecpRABCDE* gene and importantly, 90% of *K. pneumoniae* strains can produce ECP fimbriae. Ultrastructural and immunoassay analysis of *K. pneumoniae* showed that ECP can bind bacteria to each other, thus forming specific micro-colonies on cultured epithelial cells and stable biofilms on inert surfaces. ECP likely also plays an important role in cell adhesion, biofilm formation and several niche colonization, especially for isolates lacking MrkD adhesin or the entire type 3 fimbriae (Alcántar-Curiel et al., 2013).

Wu et al. (2010) found seven new fimbriae gene clusters in *K. pneumoniae*, namely *kpa*, *kpb*, *kpc*, *kpd*, *kpe*, *kpf*, and *kpg*. The loss of *kpgC* resulted in an obvious decrease in biofilm formation, adhesion to animal cells, and intestinal colonization in mice. Further, Δ*kpaC* and Δ*kpeC* mutants were also found to weaken biofilm formation and adhesion to Arabidopsis cells, respectively. The deletion of the *kpjC* usher-coding gene was shown to significantly reduce biofilm formation, while the loss of the *kpaC* usher gene was shown to only affect early and late stages of biofilm formation (Khater et al., 2015). The *kpf* gene cluster encodes type 1-like fimbriae, while the *kpfR* gene encoding the transcription inhibitor of the *kpf* gene cluster negatively regulates the expression of fimbriae. *K. pneumoniae* lacking the *kpfR* gene showed a hyperfimbriated phenotype and enhanced adhesion to epithelial host cells and biofilm formation (Gomes et al., 2020).

### 2.3. Quorum sensing (QS)

During biofilm formation, QS mediates inter-specific or intra-specific interactions through which bacterial cells communicate with each other (Bassler et al., 1993; Miller and Bassler, 2001). The QS system regulates the synthesis of fimbriae, exopolysaccharides, adhesins, and other substances through signaling molecules, thus affecting biofilm formation in bacteria (Yang et al., 2013; Gu et al., 2021). Depending on bacterial cell density, bacteria will produce and detect specific signaling molecules called auto inducers (AIs) to coordinate their gene expression (Bassler et al., 1993; Miller and Bassler, 2001). There are two main types of intercellular QS regulatory systems, namely type I and type II.

Type I QS is mainly used for intraspecific communication, which is usually related to the LuxI/LuxR system. LuxI synthetase produces N-acyl homoserine lactone (AHL) as an AI, and the LuxR transcription factor is their homologous receptor. However, *K. pneumoniae* does not produce AHL (Balestrino et al., 2005), rather encodes SdiA, which is an orphan LuxR receptor that reacts with exogenous AHL molecules produced by other bacteria (Pacheco et al., 2021). SdiA plays a repressive role in the expression of type 1 fimbriae in *K. pneumoniae*. Cells lacking SdiA regulator presents a hyperfimbriated phenotype that render the Δ*sdiA* mutant strain with a greater ability to form biofilm and agglutinate yeast cells (Pacheco et al., 2021).

**Figure 2:**
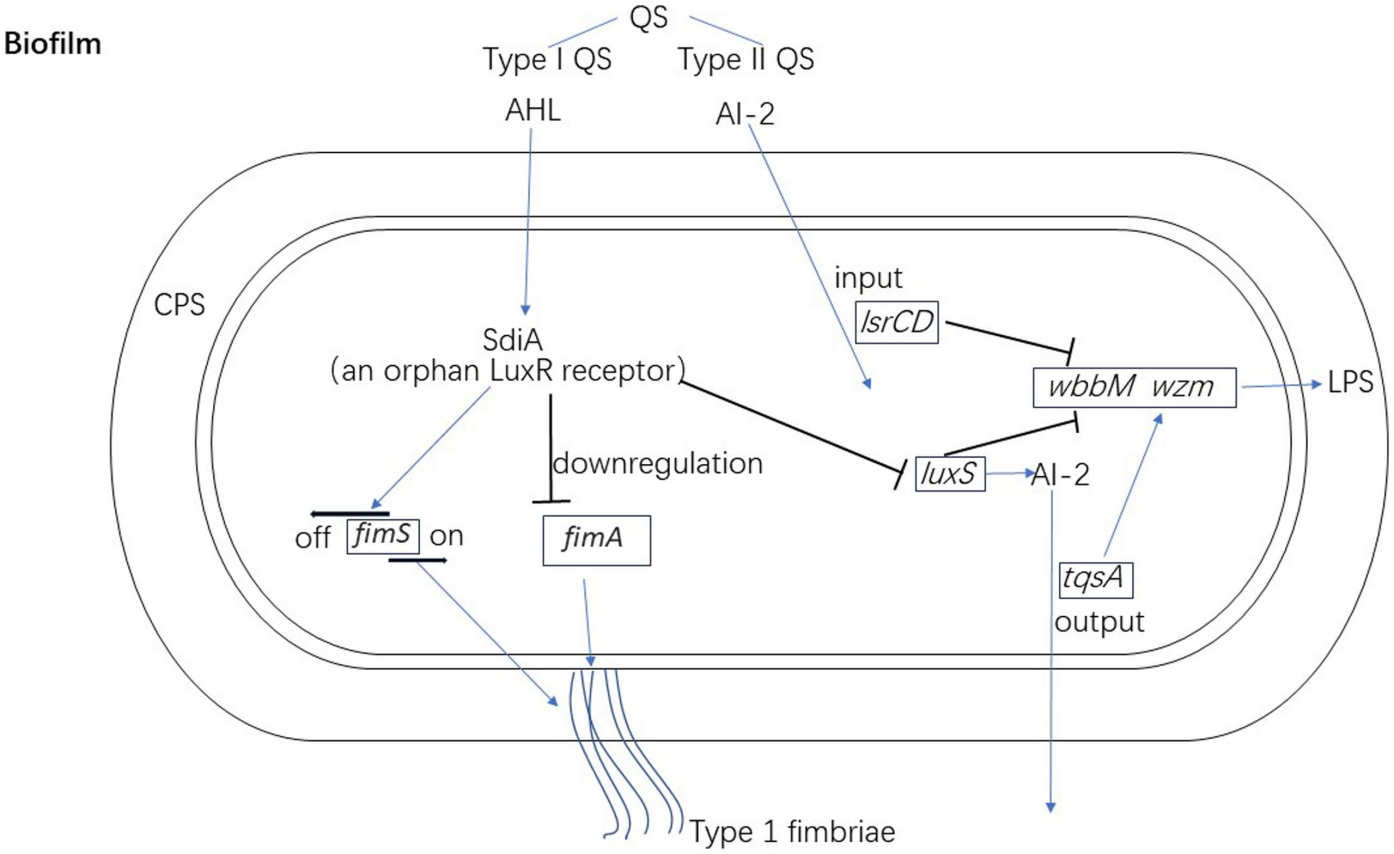
The regulation mechanisms of quorum qensing in biofilm formation of *K. pneumoniae.* *Klebsiella pneumoniae* encodes SdiA as an orphan LuxR receptor to down-regulate the expression of *fimA* (type 1 fimbriae) and *luxS*. SdiA also regulates the promoter region of the *fimS* at OFF orientation. AI-2 transport systems (*tqsA and lsrCD*) and *luxS* of *K. pneumoniae* both regulate the expression of *wbbM* and *wzm* in connection with LPS synthesis.

Type II QS has an interspecific communication function, enabling bacteria to react not only to AI-2 produced by other species, but also to their own AI-2 (Chen et al., 2002; Zhang et al., 2020). De Araujo et al. observed that *K. pneumoniae* lacking AI-2 output (Δ*tqsA*) or input (Δ*lsrCD*) systems showed an increased surface coverage after growth in dynamic micro-fermentation but decreased biofilm thickness. In addition, production of AI-2 relies on the presence of *luxS* but the biofilm structure of Δ*luxS* mutants is different. In these mutants the surface coverage rate is lower, and fewer large colonies are formed. Mutations related to *luxS* and AI-2 transport systems both induce increased expression of *wbbM* and *wzm* in connection with LPS synthesis, which indicates that QS affects biofilm formation through LPS in *K. pneumoniae* (De Araujo et al., 2010). The regulation mechanisms of the QS in biofilm formation of *K. pneumoniae* is shown in Figure 2.

## 3. Other factors affecting biofilm formation of *Klebsiella pneumoniae*

### 3.1. Nutritional condition

Nutritional conditions are also an important factor for biofilm formation. Excess nutrition may promote the planktonic growth model, while malnutrition environments are more favorable for the biofilm growth model (Stanley and Lazazzera, 2004).

Previous studies have found that high concentration of sugars (such as glucose) prohibit biofilm formation in *K. pneumoniae* and *E. coli* (Jackson et al., 2002; Sutrina et al., 2015). Glucose-rich medium inhibits the production of cyclic AMP (cAMP), a well-known second messenger that has important effects on gene regulation (Rickenberg, 1974). Furthermore, cAMP forms a homodimer (CRP-cAMP) with its signal transduction target, cAMP receptor protein (CRP), and then combines with the CRP binding site in the DNA promoter region to regulate mRNA transcription. External glucose inhibits the function of CRP-cAMP in *K. pneumoniae*. CRP indirectly regulates the expression of type 3 fimbriae through the c-di-GMP signal pathway (Lin et al., 2016). In addition, CRP mediates catabolite repression. The absence of CRP increases the concentration of c-di-GMP and reduces the activity of PDE in cells. The expression of *mrkHI* depends on c-di-GMP which in turn increases the expression of *MrkH* and *MrkI*, leading to the high expression of type 3 fimbriae. It was reported that inserting an open-reading frame containing CRP-activation domain into *K. pneumoniae* resulted in biofilm deficiency (Boddicker et al., 2006). However, other studies found that *crp* mutant *K. pneumoniae* strains could not express MrkA, the major subunit of the fimbrial shaft, which indicated that CRP was required for fimbriae production and biofilm formation (Ou et al., 2017; Panjaitan et al., 2019). These studies indicate the important regulating role of CRP in biofilm formation of *K. pneumoniae*.

Cellobiose also affects biofilm formation in *K. pneumoniae*. It has previously been shown that the *celB* deletion mutation, which leads to cellobiose deficiency, clearly decreased biofilm formation in *K. pneumoniae*. Moreover, *celB* encodes the cellobiose-specific subunit IIC of enzyme II (EIIC) of a carbohydrate phosphotransferase system (PTS, a sugar transport system in bacteria) (Wu et al., 2012). Horng et al. (2018) showed that a non-characteristic enzyme II complex homolog of PTS in *K. pneumoniae* actively regulated biofilm formation by enhancing eDNA and capsular polysaccharide production.

Different carbon sources can also affect the biofilm formation of *K. pneumoniae*. The isolates formed more robust biofilms when grown with fucose as the sole carbon source than with glucose or glycerol. It was related to the positive modulates of fucose to hypermucoviscosity of *K. pneumoniae* (Hudson et al., 2022).

The presence of bile salts can stimulate biofilm formation in *K. pneumoniae*, which is related to the production of poly-β-1,6-N-acetyl-d-glucosamine (PNAG) (Chen et al., 2014). PNAG is a common bacterial surface polysaccharide and a significant component of the biofilm EPS (Chen et al., 2020). PAGA, which is encoded by *pgaABCD* (Cywes-Bentley et al., 2013), mediates the intercellular binding of bacterial species and surface adhesion. The biofilm formation in *pgaA* mutants was shown to be significantly decreased (Wu et al., 2011). The loss of *pgaC* in *K. pneumoniae* reduces PNGA production, and significantly affects the enhancement of 1% bile salt mixture on *K. pneumoniae* biofilm (Chen et al., 2014).

Iron is indispensable in *K. pneumoniae* growth and virulence factor expression (Chhibber et al., 2013; Chen et al., 2020). A study showed that a certain concentration of iron (0.16 mM FeCl_2_) could promote biofilm formation in *K. pneumoniae* by inhibiting succinic acid. This may be due to a reduction in protein and polysaccharide expression in the biofilm EPS since succinic acid participates in pyruvate metabolism and amino acid synthesis (Liu et al., 2022). Chen et al. observed that biofilm formation was strongest when *K. pneumoniae* was cultured in LB broth supplemented with 50 μM iron. When the strain was cultured with an iron chelator, biofilm formation decreased (Chen et al., 2020). Chhibber et al. studied the biofilm formation of *K. pneumoniae* in the presence of Co [II] (iron antagonist ions) and depolymerase producing phage (degrading extracellular polysaccharides on biofilm structure). A significant reduction was observed in the growth of younger biofilms (1–3 days old) when 500 μM CoSO_4_ and 10 μM FeCl_3_ supplemented medium was used. Moreover, a complete eradication of the younger biofilms was observed when both elements were present (Chhibber et al., 2013).

### 3.2. Drugs

The use of some drugs will instead promote biofilm formation. In the presence of sub-MICs of cefotaxime, the biomass increased and was positively related to the antibiotic concentration (Hennequin et al., 2012). When CRKP strain was under antibiotic pressure, the expression of the *Psp* and *Pho* family genes [PspB-PspC complex is a pressure receptor that plays a role in molecular switch during the process of biofilm pressure response (Flores-Kim and Darwin, 2016)] was induced, thus further mediating the downstream stress responses, and compensating for the adsorption, colonization, and biofilm formation (Bowen et al., 2021). Cadavid et al. (2018) found that in *K. pneumoniae*, hydrochlorothiazide and acetaminophen could promote biofilm formation.

### 3.3. Antibiotic-resistant genes

Antibiotic-resistant genes in special plasmids can regulate the biofilm formation of *K. pneumoniae* (Maeyama et al., 2004). Multidrug-resistant (MDR) *K. pneumoniae* often forms stronger biofilms than non-MDR strains (Shadkam et al., 2021). The plasmid encoding cephalosporin enzyme was shown to obtain a transcription factor, namely *AmpR*, which was involved in upregulating capsule synthesis and antiserum killing, regulating the expression of type 3 fimbriae and biofilm formation (Hennequin et al., 2012). In addition, compared to the control strains, strong biofilm formation was found in NDM-1 producing *K. pneumoniae*. Moreover, the resistance genes *blaNDM-1* of *K. pneumoniae* were observed to be maximally up-regulated in 24 h-biofilms (Al-Bayati and Samarasinghe, 2022).

Bacterial efflux pumps are made up of transmembrane proteins, which export a variety of harmful substances including different types of antibiotics from the intracellular environment to the external environment. This process is one of the causes of MDR (Du et al., 2018). The role of efflux pumps in biofilm formation is still controversial. Tang et al. found that the efflux pump inhibitor, CCCP, had a dose-dependent effect on biofilm formation (Knight et al., 2018). In another study, researchers found that the up-regulation of the AcrAB multidrug efflux system was observed only in XDR strains with biofilm growth that could be considered an essential factor in the biofilm-forming ability in *K. pneumoniae* (Vuotto et al., 2017). In turn, biofilms have been shown to up-regulate *K. pneumoniae* efflux pump genes *acrA*, *emrB*, *oqxA,* and *qacE*Δ1 (Tang et al., 2020). However, some studies have suggested that there is no correlation between the expression of efflux pump genes (*acrA*, *kexD*, *kdeA*, *kpnEF* and *ketM*) and biofilm formation (Türkel et al., 2018).

### 3.4. Physical environment

The physical environment of bacteria will affect biofilm formation. Some physical and chemical properties of the surface on which bacteria grow may interfere with biofilm formation by damaging the initial bacterial attachment to the surface (Bos et al., 1999; Li and Logan, 2004). Biofilm production of *K. pneumoniae* decreased at 37°C compared to at 30°C, but the difference was not significant (Hostacká et al., 2010). In another experiment with 17 CRKP isolates, biofilm formation was greater at 37° C than at 25° C (Gual-de-Torrella et al., 2022). An increase in the pH of culture medium led to an increase in biofilm formation, and in *K. pneumoniae* this increased by 151–319% at pH 8.5 and by 113–177% at pH 7.5, respectively compared to at pH 5.5 (Hostacká et al., 2010).

*Klebsiella pneumoniae* growing under simulated microgravity (SMG) conditions formed a thicker biofilm than those growing under normal gravity conditions. Moreover, under SMG conditions, the cellulose production and expression of type 3 fimbriae of *K. pneumoniae* were enhanced. Therefore, *K. pneumoniae* isolated from orbital spacecrafts poses a potential threat to the health of astronauts (Wang et al., 2016).

### 3.5. Double-stranded DNA breaks

Recently, the CRISPR-Cas9 technique has been implemented to eliminate certain bacteria by use of bacteriophages or bacterial conjugation. This technique allows targeted editing of genomes by inducing double-stranded-DNA breaks (DSBs). However, a novel type of biofilm (“R-biofilm”) was found in clinical isolates of *K. pneumoniae* after DSBs. R-biofilms are mainly made up of extracellular proteins and/or DNA, which may be released by dead bacteria. In addition to bacterial SOS reaction (severe DNA damage in cells will result in SOS reaction), new signaling pathways also participate in the formation of R-biofilms. Furthermore, R-biofilms form a fixed ring or disk shape with better ductility, which can protect living bacterial cells in the body from harmful conditions such as exposure to ethanol, hydrogen peroxide, and ultraviolet radiation. The discovery of R-biofilms indicated the limited effect of the current popular Cas9-mediated sterilization tools, because the resulting DSBs may facilitate the formation of these new protective biofilms (Liu et al., 2020).

## 4. Conclusion

In the past decades we have gained considerable knowledge about the molecular mechanisms involved in the biofilm formation of *K. pneumoniae*. Similar to other bacteria, biofilm formation of *K. pneumoniae* is an adaptive response to various stressors such as nutritional deficiency, physical environment change, and drugs (especially antibiotics). Biofilm formation is not a precisely conserved process, the pattern of biofilm formation of *K. pneumoniae* is similar to other Gram-negative bacteria (Ruhal and Kataria, 2021). For example, O antigen of LPS is related to the production of capsule polysaccharide of *K. pneumoniae* and influences biofilm formation, which is common in Gram-negative bacteria (Fedtke et al., 2007; Lee et al., 2016). In general, all flagellated bacteria approach by motility and condition the surface by the secretion of polysaccharides to help cells adhere. As mentioned previously, *K. pneumoniae* use type 1 and type 3 fimbriae to adhere to surfaces (Schroll et al., 2010). *Pseudomonas aeruginosa* also use flagellar motility to reach surfaces and subsequently use type IV pili motility to crawl on surface (Zhao et al., 2013). Regulation by the two-component system via c-di-GMP are involved in the biofilm formation of most Gram-negative bacteria, including *K. pneumoniae* (Ruhal and Kataria, 2021). QS plays significant roles in biofilm formation and dispersal (Solano et al., 2014). The QS molecules are various in different bacteria. *K. pneumoniae* encodes SdiA as an AI to inhibit biofilm formation (Pacheco et al., 2021). However, *P. aeruginosa* produce AHL as an AI to influence biofilm formation (Pesci et al., 1997). Owing to the rapid spread of CRKP and hvKP, the most interesting aspect of *K. pneumoniae* biofilm formation is the impact of carbapenem-resistant plasmids or hvKP-specific virulence plasmids on biofilm formation.

**Figure 3:**
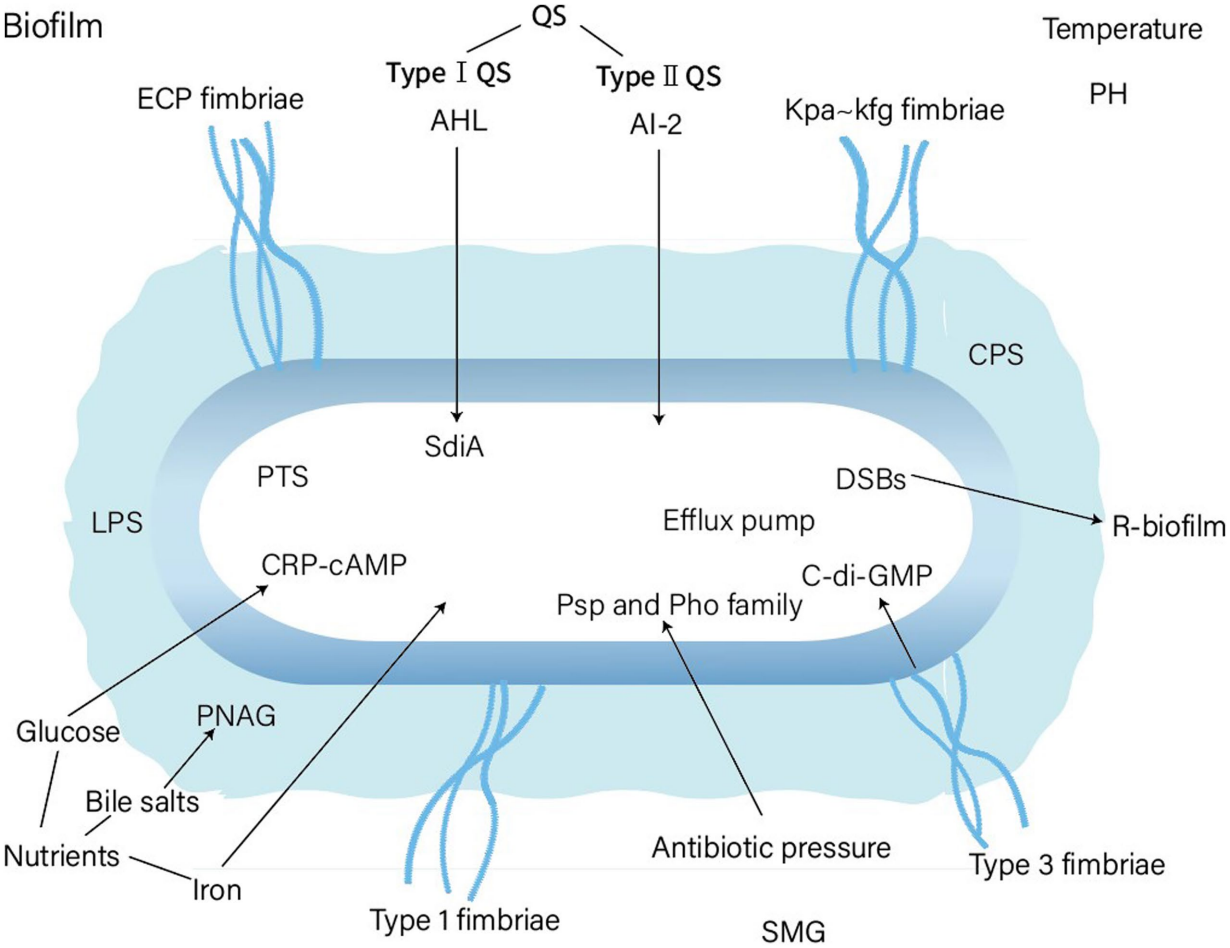
Factors affecting biofim formation of *Klebsiella pneumoniae*. PTS, phosphotransferase system; PNAG, Poly-β-1,6-N-acetyl-d-glucosamine; DSBs, double strand DNA breaks; SMG, simulated microgravity.

The various factors and genes affecting biofilm formation in *K. pneumoniae* are shown in Figure 3 and Table 1. Some controversy remains regarding certain factors in different studies. Moreover, the mechanisms by which the above factors affect the biofilm formation of *K. pneumoniae* requires further study. For instance, the role and the molecular mechanisms of the capsule of *K. pneumoniae* in biofilm formation is still unclarified. In summary, realizing the commonality and specifics of biofilm formation between *K. pneumoniae* and other bacteria will lead to a deep understanding of bacterial interactions within natural or host infection environment. Furthermore, it would be helpful to develop new therapeutic strategies for *K. pneumoniae* biofilm.

**Table 1:** Genes related to biofilm formation of *Klebsiella pneumoniae*.

Gene(s)	Explanation	Effect of knockout on biofilm formation	References
*cps gene cluster, *magA*, *wzc*, *k2A*, *wzyK2*, *rmpA*+ and *rmpA2*+	Related to the Capsul	Enhanced	Balestrino et al. (2008), Zheng et al. (2018), and Ashwath et al. (2022)
*wabG*	Involved in LPS synthesis and related to capsule	Enhanced	Hennequin et al. (2012) and Ashwath et al. (2022)
*treC*	Encodes trehalose-6-phosphate hydrolase	Enhanced	Wu et al. (2011)
*sugE*	Encodes an intima protein	Reduced	Wu et al. (2011)
*wcaG*	Related to the biosynthesis of fucose and is connected with K1 and K54 capsule types	Enhanced	Hennequin et al. (2012) and Zheng et al. (2018)
*wbaP*	Related to capsule production	Reduced	Ernst et al. (2020)
*orfX*	Negatively controlled biofilm formation by reducing CPS	Reduced	Horng et al. (2022)
*mrkA*, *mrkD*, *mrkH*, *mrkI*	Related to type 3 fimbriae	Enhanced	Di Martino et al. (2003), Jagnow and Clegg (2003), Johnson et al. (2011), Wilksch et al. (2011), Wu et al. (2012), Fang et al. (2021), and Ashwath et al. (2022)
*mrkJ*	Encodes a hypothetical phosphodiesterase	Reduced	Johnson and Clegg (2010)
*yfiRNB*	Part of an operon	Enhanced	Huertas et al. (2014)
*yjcC*	An *in vivo* expression (IVE) gene, has PDE activity	Reduced	Huang et al. (2013)
*fimH*	Relate to type 1 fimbriae	Enhanced	Ashwath et al. (2022)
*fimK*	A regulatory gene	Reduced	Rosen et al. (2008)
*kpaC*, *kpeC kpgC*, *kpjC*	Related to the corresponding fimbriae	Enhanced	Khater et al. (2015)
*kpfR*	Encodes the transcription inhibitor of the kpf gene cluster	Reduced	Gomes et al. (2020)
*wbbM* and *wzm*	The biosynthesis of O-antigen	Enhanced	Boddicker et al. (2006), Balestrino et al. (2008), and Vuotto et al. (2017)
*sdiA*	Encodes sdia, which is an orphan luxR receptor	Reduced	Pacheco et al. (2021)
*luxS*	Type 2 quorum-sensing regulatory system	Enhanced	Balestrino et al. (2005) and Vuotto et al. (2017)
*tqsA*	AI-2 output	Enhanced	De Araujo et al. (2010)
*lsrCD*	AI-2 input	Enhanced	De Araujo et al. (2010)
*celB*	Encode the putative cellobiose-specific subunit IIC of enzyme II	Enhanced	Wu et al. (2012)
*pgaABCD*	Synthesis of PNAG	Enhanced	Chen et al. (2014) and Vuotto et al. (2017)
*acrB*	Efflux pump	Enhanced	Vuotto et al. (2017)
*wcaJ*	Encodes the initiating enzyme of colanic acid synthesis and loads the first sugar (glucose-1-P) on the lipid carrier undecaprenyl phosphate	Reduced	Pal et al. (2019)
*treB*	Encodes Eitre protein	Enhanced	Wu et al. (2011)
*iutA	Aerobactin	Enhanced	Zheng et al. (2018) and Ashwath et al. (2022)
*allS	Allantoin	Enhanced	Zheng et al. (2018)
*ampR*	Regulates the synthesis of cephalosporinase DHA-1	Reduced	Hennequin et al. (2012)
*oxyR*	A lysr-type regulator	Enhanced	Hennequin and Forestier (2009)
*frwC*	Encodes EIIC-like protein, which is required for a putative fructose PTS	Reduced	Lin et al. (2018)
*KPN00353-KPN00352-KPN00351*	Encodes a putative enzyme II complex in PTS	Enhanced	Horng et al. (2018)

*Among the strains of magA (K1), aero+, rmpA+, rmpA2+, allS+, wcaG+, and iutA+, biofilm formation was more obvious than those of the strains with negative virulence factors. However, multivariate regression analysis showed that wcaG was the only independent risk factor for biofilm formation (Zheng et al., 2018).

## Author contributions

YL wrote the manuscript and searched for references. MN developed the concept and added valuable insights into the manuscript. All authors have read and agreed to the published version of the manuscript.

## Conflict of interest

The authors declare that the research was conducted in the absence of any commercial or financial relationships that could be construed as a potential conflict of interest.

## Publisher’s note

All claims expressed in this article are solely those of the authors and do not necessarily represent those of their affiliated organizations, or those of the publisher, the editors and the reviewers. Any product that may be evaluated in this article, or claim that may be made by its manufacturer, is not guaranteed or endorsed by the publisher.
